# When to Eat and When to Play: Variations in Recess and Lunch Scheduling Within a State 40‐Min Recess Policy

**DOI:** 10.1111/josh.70055

**Published:** 2025-08-08

**Authors:** Erin K. Howie, Samantha M. Harden, Juan Lemus, Brett Stone, Daheia J. Barr‐Anderson, Christopher R. Long

**Affiliations:** ^1^ Department of Health, Human Performance and Recreation University of Arkansas Fayetteville Arkansas USA; ^2^ Department of Human Nutrition, Foods, and Exercise Virginia Tech Blacksburg Virginia USA; ^3^ Department of Obstetrics/Gynecology, Department of Family and Community Medicine Virginia Tech Carilion School of Medicine Roanoke Virginia USA; ^4^ Department of Health and Physical Education Arkansas Tech University Russellville Arkansas USA; ^5^ Department of Applied Physiology, Health, and Clinical Sciences University of North Carolina at Charlotte Charlotte North Carolina USA; ^6^ Center for Nutrition & Health Impact Omaha Nebraska USA

## Abstract

**Background:**

Little is known on the implementation of school recess policies to explore the policy‐to‐practice gap and to ultimately promote quality recess for all students. The purpose was to determine recess scheduling in a state requiring 40 min of daily recess.

**Methods:**

A cross‐sectional document analysis of public elementary schools' recess schedules from the 2023 to 2024 school year (*n* = 526 available schools). Schedules by grade level were compared between school grades, rurality, and minority enrollment.

**Results:**

Schedules were obtained from 113 (*n* = 535 grade‐level schedules) schools. Overall, 51.4% of grades received two daily recess periods. The average total duration of recess was 34.4 (SD 11.8) minutes. A total of 43% of grades had recess before lunch. Upper grades had a shorter recess duration compared to lower grades (*B* −1.2 min, *p* = 0.009) and were more likely to have their first recess in the afternoon (OR 4.8, *p* < 0.001). Urban (*p* = 0.017) and high minority enrollment (*p* = 0.020) schools were more likely to have recess immediately following lunch.

**Implications for School Health Policy, Practice, and Equity:**

Schools should consider providing equal opportunities for recess duration and scheduling across grades and consider options for recess before lunch.

**Conclusions:**

Recess scheduling varies in frequency, duration, and timing within one state‐level recess requirement.

Recess, unstructured play occurring outdoors when possible, has multiple benefits for children including physical, mental, and social benefits [1]. Recess has also been associated with several positive educational outcomes including improved classroom behavior, cognition, and academic achievement [2]. The benefits of recess are likely due to several potential mechanisms including physical activity [3], unstructured and social play [4], outdoor exposure [5], and relationships with lunch nutrition [6, 7, 8]. Together, these unique characteristics of recess support whole‐child development [9].

In recognition of the importance of recess, the Centers for Disease Control and Prevention (CDC) makes several recommendations to schools for scheduling recess in the U.S. [10] For minimum recess duration, the current recommendation is a minimum of 20 min daily, but there is limited evidence supporting this specific duration of 20 min. Recent evidence exists that a longer duration might be needed for better health outcomes [11, 12]. Current state recess policies vary widely from requiring 15 min of daily recess in Louisiana to 40 min in Arkansas; yet little is known on how these varying recess policies are implemented at the school level [13]. A better understanding of “how” a policy is effectively implemented as opposed to the specific “what” of the policy can help to bridge the policy‐to‐practice gap [14]. Importantly, none of these policies make recommendations for the frequency, duration, and timing scheduling of recess periods, except Arizona, which requires two recess periods. This is likely due to a lack of evidence supporting specific scheduling. A better understanding of the implementation of recess policies can help to inform effective and feasible future recess policies and practices, as well as future research studies to examine the effects of varying recess schedules as a result of these policies.

## 1 Scheduling of Recess

Research on the scheduling of recess, though limited, suggests that the timing of recess matters for student outcomes. Two studies, one in Arizona and one in Kentucky, studied the differences in one 15‐min recess versus two 15‐min recesses, with more recess increasing physical activity [15], math achievement and discipline referrals [16]. Importantly, they found that the additional recess period may have reduced some of the discipline disparities between Black and White students. Additionally, the number of discipline referrals increased with the passing of time from a recess period [17], meaning there were more behavioral problems the longer after a recess period. This suggests that shorter durations between recesses may reduce behavioral issues in the classroom. When specifically examining the timing of recess, Pelligrini et al. manipulated the timing of recess [18]. Students in a small study either went to recess at 10 or 10:30 a.m., resulting in increased physical activity and social interaction after the delayed recess, suggesting a rebound effect to play deprivation. Thus, while a greater time between recess periods might be deleterious to classroom attention, it may result in higher physical activity; however, the evidence is limited to these few small studies. It is unknown how state recess policies affect recess scheduling at the school level.

## 2 Scheduling of Lunch

In addition to recess recommendations, the CDC recommends a minimum of 20 min of seat time for lunch, with research showing that longer lunch durations result in improved mealtime and health outcomes [19, 20]. A 30‐min scheduled lunch period is recommended to achieve 20 min of seat time, and less than half of schools in the U.S. reported a minimum of 30 min of scheduled lunch [21]. In addition to the minimum duration of lunch and recess, the CDC also recommends that morning recesses should be scheduled before lunch but does not specify the recommended timing between recess and lunch [10]. This variation in scheduling is of importance because recess before lunch revealed positive benefits such as reduced plate waste, increased food and milk consumption as well as an improvement in overall lunchtime environment, student behavior, and classroom concentration [6, 7, 8]. Less is known about the impact of lunch scheduling on the physical activity of students during recess [22]. Current practices on the joint scheduling of lunch and recess are unknown.

## 3 Importance of Sociodemographic and Geographic Factors

Importantly, these effects of recess scheduling may differ by gender, demographics, and geographic locations. Policies may be implemented differently based on available resources. Students who were Black or Hispanic, lived in an urban area, lived in the South, went to a public school, or were from lower socioeconomic backgrounds were more likely to receive no recess compared to some recess [23]. There is additional evidence that recess is implemented unequally across geographical and demographic factors [24]. Children in rural schools and minority populations have higher rates of physical inactivity and associated chronic conditions [25, 26], making them a priority for providing quality school‐based physical activity opportunities. Thus, to ensure that all children are receiving equal recess opportunities and potentially receiving the maximal benefits from recess, a better understanding of how recess is currently being implemented is needed.

Therefore, to inform the translation of education policy to meaningful practice, the purpose of the current study was to describe the scheduling of recess, including frequency, duration, timing, and relation to lunch across schools in Arkansas, a state that requires 40 min of daily recess. Secondly, we compared the patterns of recess by grade level, rurality, and school demographics. This will provide the first detailed implementation evidence of how a state‐level recess policy requiring a minimum duration of recess is scheduled.

## 4 Methods

### 4.1 Study Design

We conducted a cross‐sectional, document analysis of recess schedules. Recess schedules were collected from schools in Arkansas as part of a larger study on the implementation of current recess policy in the state [24]. A multi‐pronged audit utilizing web searches, school surveys, and phone calls to school offices was conducted from March to July 2024 of the 2023–2024 academic school year. The study was determined to be exempt by the university Institutional Review Board.

### 4.2 Participants

There were 526 eligible public and charter elementary schools in Arkansas during the 2023–2024 school year. Schools were eligible if they were public, including open enrollment charter schools, and classified as elementary with grades kindergarten through 5th grade. Schools were excluded if they were virtual or hybrid schools, preschools, or only included middle and high school grades (6th grade and above). Arkansas has a current recess requirement of 40 min per day for elementary schools (Arkansas Code Title 6. Education § 6‐16‐102) This recess requirement was implemented in the 2019–2020 school year.

### 4.3 Measures

Research staff conducted a multipronged audit of all elementary schools. This study utilized three methods to collect recess implementation data to ensure completeness of data [27].

#### 4.3.1 Online Search

The first method was an online search to collate publicly available recess bell schedules, collected in March 2024. Using keywords including'bell schedul', “Arkansas”, and “elementary school”, research assistants conducted a systematic search of school websites to identify available recess schedules; wellness policies and parent handbooks were also searched for information.

#### 4.3.2 School Survey

The second method utilized an online survey that was sent to all school principals and physical education teachers when contact information was available; participants could upload a copy of their recess schedules. The survey was initially emailed in late March/early April; a second follow‐up email was sent in mid‐April, and a final email was sent after the end of school in late June 2024.

#### 4.3.3 School Phone Calls

For the third method, for schools where online schedules were not available and did not respond to the survey, research assistants called school offices to collect available information on recess provision, and participants could either complete the online survey or email a copy of their recess schedules. During school business hours, research assistants called the primary school phone number listed online and read a prepared script. If the staff who answered the phone could not provide the answers, they either forwarded the call, provided additional contact information, or were emailed the link to the online survey. Schools were contacted up to two times. Initial phone calls were made in late April/early May, with follow‐up calls made in late May 2024.

### 4.4 Recess and Lunch Scheduling Extraction

Original recess schedules obtained from each source were saved electronically. Research assistants extracted recess and lunch start and stop times by grade level, as all schools schedule recess periods by grade level (e.g., all third graders have recess at the same time). Recess start and stop times were entered by grade level into an Excel worksheet. Total recess duration, inter‐recess duration as the time between the end of the first recess and the start of the second recess, lunch duration, and time between the end of recess and the start of lunch were calculated from reported start and stop times. The total amount of recess was obtained from either recess schedules, online survey, or phone calls and used to determine if the school was meeting recess guidelines [24].

### 4.5 School Characteristics

Urban/rural status was determined by county categorization from the Office of Management and Budget and the Federal Office of Rural Health Policy [28]. Demographic data, student enrollment, and enrollment by race for the 2023–2024 school year was obtained from the Arkansas Department of Education Data Center [29]. To examine school racial differences in recess provision, differences in recess scheduling by total minority student enrollment were explored. To capture schools with a high percentage of minority students, a 25% cutoff was used to dichotomize schools due to perceived cultural norms and perceptions with substantial white or minority enrollments [30]. This cutoff has been previously used to describe schools with higher minority enrollment [31, 32].

Additional school information was obtained from the most recent Office for Education Policy report (2022–2023 school year) [33]. School characteristics included grade levels: lower including PreK‐2nd grade only, upper including 3rd grade and higher, and mixed included both lower and upper elementary grades. Schools that included both grade ranges (e.g., K‐6) were described as mixed grade, with grade levels beyond kindergarten (e.g., preK) or 6th grade (e.g., 8th grade) not included in the current analysis. Additional school factors were used to describe participating schools. School economic disadvantage was reported as the percent of students eligible for free‐or‐reduced lunch (%FRL). Standardized test scores were used as measures of school academic achievement, with both weighted achievement and growth scores indicating annual change in student performance.

### 4.6 Statistical Analysis

Descriptive characteristics of the schools are reported and compared between those with and without recess schedules using *t*‐tests or chi‐squared tests. Some schedules did not include all information, including start and stop times of recess or lunch times (e.g., only had start time). All available information is reported along with available n's reported for each variable.

As almost all schools report and schedule recess by grade level based on the recess schedules obtained, the unit of analysis was grade. Multilevel linear or logistic regression models were used to compare recess characteristics between grade levels, rurality, and minority enrollment, with multiple grades clustered within school. As models were exploratory, they are unadjusted for further covariates. Due to fewer schools with 5th and 6th grades, analyses by grade were categorized into schools including lower (K‐2nd) and upper (3rd–6th grades). Recess timing was categorized into early recess (before 10 a.m.), midmorning recess (10 a.m. to before 12 p.m.), and afternoon recess (12 p.m. or later) with the majority of elementary schools in Arkansas having start times between 7:45 and 8:15 a.m. and end times of 2:45 to 3:10 p.m. All analyses were conducted using Stata IC/14.2 (College Station, TX) and statistical significance was considered at *p* < 0.05.

## 5 Results

School schedules were obtained from 110 (*n* = 578 grade‐level schedule) schools in Arkansas. As part of the larger study, some recess information was obtained from 384 schools [24], however, providing a copy of a recess schedule with timings was optional in the school survey or school phone call. Schools with recess schedules were compared to those without recess schedules in Table 1. There were no statistical differences in school enrollment, racial demographics, school economic disadvantage, rurality, or school academic achievement between schools with and without recess schedules, except for the percentage of meeting recess recommendations. Of the included schools, 35 (34.3%) were in rural counties and 61 (59.8%) had higher than 25% minority enrollment. There were no differences in minority enrollment between rural and urban schools (rural 34.3% minority enrollment versus 43.0% in urban schools; *t*‐test, *p* = 0.150).

**TABLE 1:** Description of schools with and without recess schedules.

	Schools with recess schedules, *n* = 110	Schools without recess schedules, *n* = 416	*p*‐value	Excluding incomplete online schedules (*n* = 102)	Schools without recess schedules, (*n* = 424)	*p*‐value
School Enrollment, *M* (SD)	382.7 (168.4)	402.3 (156.2)	0.252	378.3 (171.7)	403.0 (155.5)	0.159
Grades in School, *N* (%)						
*Lower*	6 (5.5%)	37 (8.9%)	0.408	5 (4.9%)	38 (9.0%)	0.210
*Upper*	9 (8.2%)	41 (9.9%)		7 (6.9%)	43 (10.1%)	
*Mixed*	95 (86.4%)	338 (81.3%)		90 (88.2%)	343 (80.9%)	
Enrollment by Race, *M* (SD)						
*% Black*	17.1 (24.1)	21.1 (28.4)	0.187	17. (24.1)	21.1 (28.4)	0.187
*% Hispanic*	15.0 (16.4)	12.5 (14.5)	0.133	15.0 (16.4)	12.5 (14.5)	0.133
*% White*	59.1 (28.9)	58.2 (29.9)	0.790	59.1 (28.9)	58.2 (29.9)	0.790
*% Minority* ^a^	40.0 (29.0)	40.9 (29.6)	0.788	40.0 (29.0)	40.9 (29.6)	0.788
% Schools with > 25% minority enrollment	66 (60.0%)	250 (61.3%)	0.808	61 (59.8%)	250 (61.3%)	0.785
% Students receiving FRL, *M* (SD)^a^	67.3 (20.0)	64.2 (20.0)	0.162	67.3 (20.0)	64.2 (20.0)	0.162
Schools in Rural Counties, *N* (%)	36 (32.7%)	169 (40.6%)	0.131	35 (34.3%)	170 (40.1%)	0.282
Region, *N* (%)			0.099			0.260
*1—Northwest*	39 (35.5%)	149 (35.8%)		39 (38.2%)	149 (35.1%)	
*2—Northeast*	15 (13.6%)	95 (22.8%)		14 (13.7%)	96 (22.6%)	
*3—Central*	40 (36.4%)	104 (25.0%)		34 (33.3%)	110 (25.9%)	
*4—Southwest*	10 (9.1%)	42 (10.1%)		10 (9.8%)	42 (9.9%)	
*5—Southeast*	6 (5.5%)	26 (6.3%)		5 (4.9%)	27 (6.4%)	
Charter School^a^, *N* (%)	3 (2.7%)	15 (3.7%)	0.630	3 (2.9%)	15 (3.6%)	0.743
Weighted Achievement^a^, *M* (SD)	55.3 (15.3)	56.9 (15.3)	0.347	55.3 (15.3)	56.9 (15.3)	0.347
Value‐added Growth Score^a^, *M* (SD)	80.6 (3.5)	80.7 (3.1)	0.829	80.6 (3.5)	80.7 (3.1)	0.829
Meeting recess recommendations (*n* = 384)	83 (76.2%)	233 (84.7%)	**0**.**047**	83 (82.2%)	233 (82.3%)	0.972

^a^ Information obtained from the Office for Education policy reports and 8 schools did not have school achievement %FRL, %minority, or academic achievement data.

When the method of recess schedule obtainment was examined, schedules obtained online without verification from another source were significantly different in those meeting 40‐min recess requirements (39% of online‐only schedules vs. 85% of either survey or phone call schedules meeting recess requirements). Online schedules were examined, and several were suspected of not being complete recess schedules that did not report all recess periods. Schools with online schedules that reported minimal information with only one recess less than 30 min immediately following lunch and no other scheduled recesses were excluded (*n* = 8) from the recess analysis but included for the lunch and timing information when available. Sensitivity analyses were conducted for the complete sample and those excluded for incomplete online schedules, with no differences in the overall findings. Results excluding those with incomplete online schedules are reported for completeness, with all schools included for reported lunch schedules resulting in *n* = 547 grade‐level recess schedules for *n* = 102 schools included in the final analyses.

### 5.1 Recess Scheduling Frequency, Duration, and Timing

Characteristics of recess schedules are reported in Table 2 and recess schedules reported by each grade individually in (Supplemental Table 1). Overall, the majority (50.6%) of students received two recess periods per day, with 47.2% having one recess period and 2.2% receiving three. This differed between grades (*p* < 0.001); upper grades were more likely to receive one recess period (55.1% of upper grades compared to 39.6% of lower grades) and lower grades were more likely to receive two recess periods (58.2% of lower grades compared to 42.7% of upper grades).

**TABLE 2:** Description of recess scheduling characteristics by grade, *n* = 547 grades (*n* = 102 schools).

Recess characteristics (*n* = grades included)	Total (*n* = 547)	Lower grades (*n* = 280)	Upper grades (*n* = 267)	*p*‐value from *t* test or chi‐squared
Number of recess periods (*n* = 533)				**< 0.001**
*1*	258 (47.2%)	111 (39.6%)	147 (55.1%)	
*2*	277 (50.6%)	163 (58.2%)	114 (42.7%)	
*3*	12 (2.2%)	6 (2.1%)	6 (2.3%)	
Combined Lunch Recess	42 (7.7%)	22 (7.9%)	20 (7.5%)	0.872
Recess Patterns (*n* = 547)				**0.007**
*One 40‐min recess*	88 (16.1%)	37 (13.2%)	51 (19.1%)	
*Two 20‐min recesses*	195 (35.7%)	118 (42.1%)	77 (28.8%)	
*One 30‐min recess*	88 (16.1%)	38 (13.6%)	50 (18.7%)	
*Other*	176 (32.2%)	87 (31.1%)	89 (33.3%)	
Total recess duration (*n* = 547)	34.4 (11.8)	35.1 (11.7)	33.7 (11.8)	0.171
Duration of recess period (1 recess period) (*n* = 232)	32.8 (8.5)	32.6 (8.6)	33.0 (8.5)	0.686
Duration of 1st recess period (2+ recess periods) (*n* = 282)	20.5 (4.1)	20.7 (4.1)	20.2 (4.2)	0.243
Duration of 2nd recess period (2+ recess periods) (*n* = 264)	20.2 (4.4)	20.2 (4.8)	20.1 (3.8)	0.881
Duration between recess periods (*n* = 253)	145.1 (64.1)	148.2 (60.7)	140.8 (68.7)	0.365
Lunch duration (*n* = 345)	27.9 (5.7)	28.4 (5.8)	27.4 (5.5)	0.122
Recess before lunch (*n* = 391 grades)	168 (43.0%)	82 (40.6%)	84 (45.2%)	0.662
Recess immediately after lunch (*n* = 345 grades)	208 (60.3%)	104 (57.8%)	104 (63.0%)	0.319
First recess timing (*n* = 517)				**< 0.001**
*Early morning* (*before 10 a.m*.)	103 (19.9%)	49 (19.6%)	54 (21.3%)	
*Late morning* (*10 a.m. until noon*)	257 (49.7%)	164 (62.1%)	93 (36.8%)	
*Afternoon* (*Noon or later*)	157 (30.4%)	51 (19.3%)	106 (41.9%)	

The average total duration of recess was 34.4 (SD 11.8) minutes. For grades with one recess period, the average recess duration was 32.8 (SD 8.5) minutes. The average duration of recess periods for grades with two recess periods was 20.5 (4.1) minutes for the first recess and 20.2 (4.4) minutes for the second recess. The average lunch duration was 27.9 (5.7) minutes.

When examining the timing of recess overall, 43.0% of grades had recesses before lunch. Of grades with one recess period, 22.4% had recess before lunch, while 59.8% of those with two or more recess periods had a recess before lunch. For grades with lunch before recess, the average time between the end of the recess and start of lunch was 64.9 (SD 59.9) minutes, with 31 grades having lunch immediately following recess and 56 grades having lunch within 30 min of the end of their recess. A small percentage, 7.7%, had combined, undefined lunch and recess periods. Lower grades were more likely to have a late morning recess (10 a.m. to noon) compared to upper grades who most commonly had an afternoon recess (noon or later).

For grades with two recess periods, the average duration between recess periods was 145.1 (64.1) minutes with a range of 30–365 min.

### 5.2 Comparison of Recess Scheduling by Grades, School Rurality, and School Racial Demographics

**FIGURE 1:**
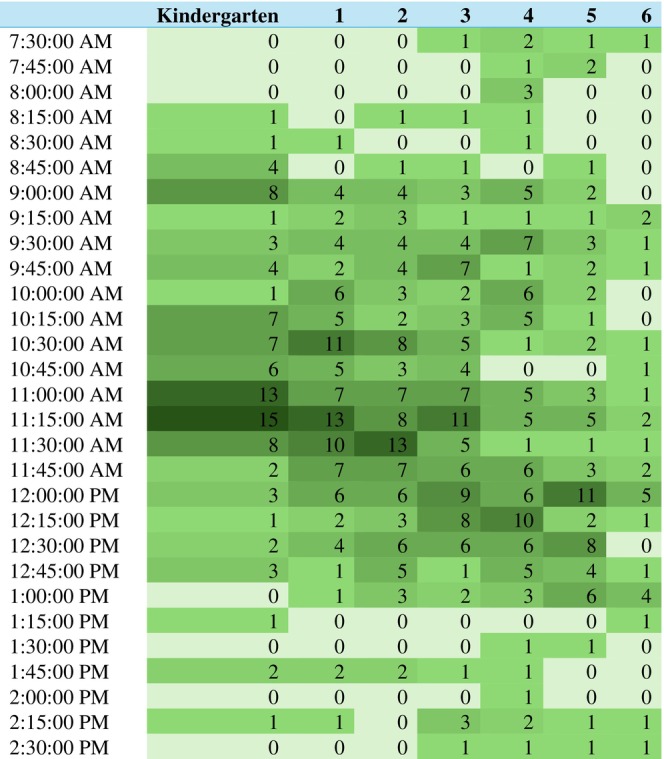
Recess start times for first recess period, all grades.

**FIGURE 2:**
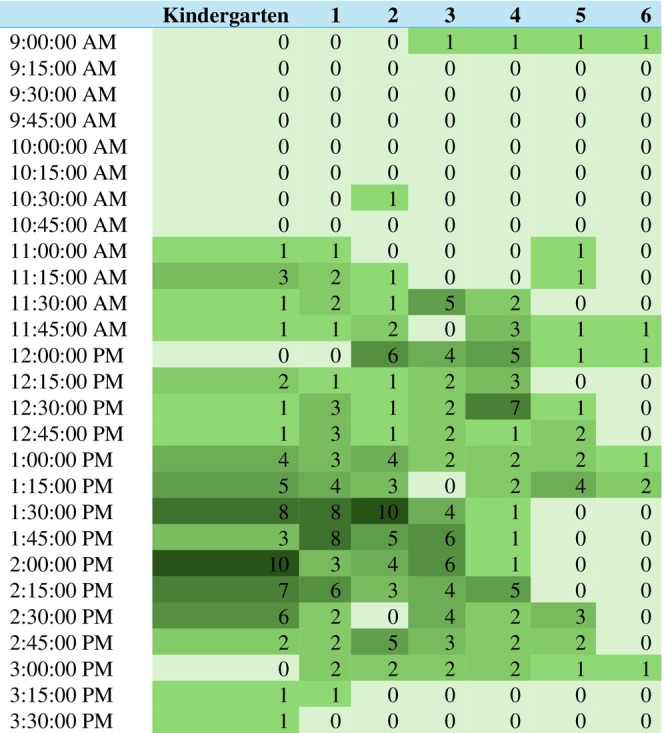
Recess start times for the second recess period (only grades with 2+ recess periods).

From multi‐level models accounting for the clustering of grades within schools, upper grades had a slightly shorter recess duration (B −1.2 min, *p* = 0.009) and were more likely to have their first recess in the afternoon (OR 4.8, *p* < 0.001) as shown in Table 3. The timing of recess start times by grade is shown in Figures 1 and 2.

**TABLE 3:** Multi‐level models for the effect of grade, rurality and school demographics accounting for clustering of grades within schools, lower grades is reference.

	Upper versus Lower grades			Rural versus Urban schools			High minority versus low minority student enrollment		
	B (SE) or OR (SE)	95% CI	*p*‐value	B (SE) or OR (SE)	95% CI	*p*‐value	B (SE) or OR (SE)	95% CI	*p*‐value
Total recess duration (*n* = 547)	−1.2 (0.5)	−2.1, −0.3	**0.009**	3.5 (2.2)	−0.8, 7.9	0.107	−4.1 (2.1)	−8.3, 0.04	0.052
Duration of recess period (1 recess period) (*n* = 232)	0.5 (0.5)	−0.4, 1.4	0.308	2.8 (2.2)	−1.5, 7.1	0.197	−2.4 (2.2)	−6.8, 2.0	0.288
Duration of 1st recess period (2+ recess periods) (*n* = 282)	−0.27 (0.3)	−0.9, 0.4	0.424	1.5 (0.9)	−0.2, 3.2	0.100	−0.3 (0.8)	−1.9, 1.4	0.755
Duration of 2nd recess period (2+ recess periods) (*n* = 264)	0.1 (0.5)	−0.8, 1.0	0.773	−0.9 (0.9)	−2.7, 0.8	0.294	0.2 (0.8)	−1.5, 1.8	0.837
Duration between recess periods (*n* = 257)	−5.8 (6.6)	−18.7, 7.0	0.372	10.8 (13.8)	−16.2, 37.9	0.269	−1.9 (13.2)	−27.7, 24.0	0.886
Lunch duration (*n* = 343)	0.002 (0.8)	−1.5, 1.5	0.998	1.6 (1.5)	−1.3, 4.5	0.276	−1.1 (2.1)	−5.1, 3.0	0.612
Recess before lunch (*n* = 388 grades)	OR 1.4 (0.4)	0.7, 2.6	0.347	OR 0.7 (0.6)	0.1, 4.0	0.662	OR 0.8 (0.7)	0.1, 4.1	0.740
Recess immediately after lunch (*n* = 345 grades)	OR 1.8 (0.7)	0.8, 4.1	0.130	OR 0.06 (0.1)	0.01, 0.6	**0.017**	OR 15.7 (18.6)	1.6, 159.3)	**0.020**
First recess timing (afternoon vs. morning) (*n* = 517)	OR 4.8 (1.2)	2.9, 7.9	**< 0.001**	OR 1.1 (0.4)	0.5, 2.3	0.764	OR 1.2 (0.4)	0.6, 2.5)	0.529

*Note: n* indicates available grades with information include in the specific analyses, all available information was used.

The only difference in recess scheduling by school geography and demographics was grades in urban (*p* = 0.017) and high minority enrollment (*p* = 0.020) schools were more likely to have recess immediately following lunch, as shown in Table 3. There were no other differences in recess scheduling characteristics, including total recess duration, lunch duration, or the timing of first recess between rural and urban or high and low minority schools. When both minority and rural status are included in the model, only rural status remains significant (*p* = 0.037).

## 6 Discussion

This study examined the detailed scheduling of recess within the implementation of a statewide 40‐min minimum recess requirement. Most grades with available recess schedules had two scheduled recess periods; however, less than half of grades had a recess period scheduled before lunch. There were differences in recess schedules between upper and lower grades, with upper grades receiving slightly less recess and more likely to have their first recess in the afternoon. Additionally, grades in urban and higher minority student enrollment were more likely to have recess scheduled immediately after lunch.

Of the recess schedules obtained, the average total recess duration was slightly less than the 40‐min requirement. This suggests that all students are not receiving 40 min of unstructured recess play per day, highlighting a policy‐to‐practice gap. Our previous research of recess in Arkansas reports high overall compliance with the 40‐min recess requirement [24]. Grades with one scheduled recess period average 33 min per day, while the total average of grades with two scheduled recess periods was 41 min. This suggests that schools with two recess periods are more likely to provide the opportunity for 40 min of play, and future state policies may want to recommend providing two recess periods per day. Creating evidence‐based policies, such as recess policies that have shown to increase quality recess time, may help to improve implementation fidelity. Additionally, more guidance from education administration or educator stakeholder collectives (i.e., school cooperatives, teacher training) on specific implementation strategies to achieve policy goals may help to bridge the policy‐to‐practice gap [14].

The current study found that a small majority of schools implement the 40‐min recess period as two recess periods, but there is wide variation in the specific scheduling, frequency, and duration. This is the first study to our knowledge to report detailed recess implementation of a state‐level recess policy. A study in Arizona, a state that requires two recess periods per day of unspecified duration, found that of 171 schools out of over 1300 elementary schools statewide responding to a survey in Fall 2021, 60% offered two or more recesses for grade three students, while 14% offered less than two recesses. Approximately 26% of respondents did not know or did not respond to the question regarding recess frequency [15]. In their national U.S. survey of schools in 2019, Tsai et al. only asked about the total daily duration and frequency of recess, without more detailed information about the specific scheduling [34]. Less recently, a U.S. study by Barros et al. defined the combined duration and frequency of recess into categories and found that 30% of 8–9‐year‐olds had none‐to‐little recess, while only 20% had more than 30 min with at least two recess periods a day [23].

When examining more specific scheduling patterns of recess in the current study, approximately one third of grades implemented two 20‐min recess periods, but there was wide variation in exactly how the total duration was distributed. There was also a wide variety in the recess period duration, with 17% of grades having a longer 40‐min recess period, 30% having 20‐min recess periods, and 38% having some other combination. The wide variation in the implementation of recess suggests that it may be important to allow schools local control to schedule recess periods depending on school staffing, specials requirements (when P.E. teachers are available), and space resources [35]. For example, schools with larger or multiple playgrounds may be able to have several grades scheduled for recess simultaneously. However, there may be differential effects on student health and learning from these different recess patterns, as previous research on the acute cognitive effects of physical activity suggests that the duration of activity may influence educational outcomes [36]. More research is needed to examine how these different recess durations and frequencies influence students. Anecdotally, several schools that participated in the survey verbally or within open‐ended survey questions were planning to switch recess period frequency for the following school year, both switching from two recess periods to one recess period and vice versa. Longitudinal studies can capitalize on these natural experiments of changes in recess scheduling on student health and education outcomes. Qualitative studies are needed to better understand the decision‐making process behind these changes in scheduling.

Another factor that may influence educational outcomes and recess physical activity is the specific timing of recess and duration between recess periods or physical activity breaks. Interestingly, a minority of grades had a recess scheduled before lunch, and upper grades often did not have a recess until the afternoon period. Lower grades consistently had recess and lunch scheduled earlier in the day. It is unknown if there is evidence to support this grade‐level practice. Average school start times for grades in the current study were 7:45–8:15 a.m. and schools with earlier or later start times might have differences in recess timing. Research suggests that adolescents have different circadian needs such as later arousal times [37] which may potentially support later lunch and recess for older children, but more evidence is needed. Another potential implication of consistent patterns of lower grades receiving earlier recess times may lead to more indoor recess time for lower grades due to weather such as too cold weather or upper grades exposed to higher temperatures depending on the local climate. As outdoor time is consistently associated with higher levels of physical activity [38], the impact of recess scheduling and the interaction of weather on quality of recess needs to be explored.

The average duration between recess periods in the current study was close to two and a half hours with a wide variation, and as mentioned, some upper grades did not receive recess until the afternoon. Evidence suggests that the longer duration between recess periods may lead to more behavioral issues occurring, with discipline referrals occurring on average 100 min following a recess period [17]. This is in line with play deprivation theory where children may experience negative outcomes with restriction and extended delay in play opportunities [18]. Thus, future research is needed to better understand the differential acute effects of these recess patterns on student outcomes, and additional policy recommendations to reduce the timing between play opportunities may be needed.

One important aspect of recess timing may be its juxtaposition with lunch. The CDC recommends a morning recess before lunch, due to the suggested benefits for eating behaviors during lunch. The current study found that the majority of students do not get recess before lunch. Those with two recess periods are more likely to get a morning recess before lunch, but still only 60% of grades with two recess periods had one period before lunch. In 2001, only 4.6% of elementary schools nationally within the U.S. had all grades with recess before lunch, while 42.4% had all of their grades with recess after lunch [39] which is similar to the 49% of grades having recess immediately after lunch in the current study over 20 years later. If state policymakers would like to encourage schools to follow the CDC recommendations, then stipulating one recess before lunch or recommending two recess periods within the policy may make it more likely. States with laws addressing minimum lunch duration are more likely to provide a minimum of 30 min of lunch [40], indicating that state‐level lunch policies have the ability to influence school‐level practices. Policies or implementation recommendations may want to continue to specifically encourage schools to schedule recess before lunch to ensure this practice is implemented [41]. Interestingly, urban and high minority student enrollment schools were more likely to have recess immediately after lunch, which could be tied to specific lunch funding programs, but more detailed examination of this is needed to explain the differences.

The “Recess before Lunch” movement recommends recess before lunch but no research has examined if the benefits are dependent on the time between recess and lunch, with assumptions that recess should be immediately before lunch [42]. In the current study, of the 43% of grades with a recess before lunch, the average time between recess and lunch was over an hour, and only 31 grades had recess immediately before lunch. More detailed research is needed on the interactions between recess, snack time, and lunch timing with student outcomes.

The current study provided an in‐depth analysis of recess schedules in one state, which limits generalizability; however, it provides an in‐depth case study for internal validity. The 102 schedules included in the analyses represent 20% of public elementary schools in Arkansas; however, there were no differences in school demographics or academic achievement between those with recess schedules and those without. Schools and stakeholders, including the Department of Education, are reluctant to release full bell schedules, likely due to safety and privacy concerns; thus, the current study represents a robust sample in the context of education. It did not consider the interrelationship between physical education and recess; however, the state requirement for physical education was only 40 min of physical education weekly; thus, it would likely have limited impact on daily physical activity, except for 1 day a week.

## 7 Implications for School Health Policy, Practice, and Equity

This study provides examples of various ways in which a 40‐min state recess requirement can be implemented; schools may want to consider alternative options for achieving the 40‐min requirement that utilize their staff capacity and physical resources. As the minority of schools are providing recess before lunch, principals may want to consider options such as two recess periods to provide more opportunities for unstructured play throughout the day, with consideration given to urban and high minority student enrollment schools that are more likely to have recess immediately after lunch and upper grades that often have later recess periods.

## 8 Conclusions

This study provides a detailed implementation analysis of a state recess policy, achieving the purpose of describing the frequency, duration, and timing of recess and its relation to lunch within a state with a 40‐min recess requirement. The study, the first to our knowledge examining the specific implementation of a state recess policy, found variations in how recess was implemented, including recess frequency, duration, and timing. Second, the study also found differences in scheduling by grade and school characteristics. Future longitudinal and experimental research is needed to test the effects of these different recess schedules on student outcomes to better inform state policies on optimal recess policy. Additionally, as more becomes known about the benefits of particular schedules, implementation guides and resources should be created to help schools schedule and implement policies that achieve the intended goals of healthier, happier, and ready‐toto‐learn students.

## Conflicts of Interest

The authors declare no conflicts of interest.

## Supporting information


Data S1.

## Data Availability

The data that support the findings of this study are available from the corresponding author upon reasonable request.
