# Supplementary material for: When to Eat and When to Play: Variations in Recess and Lunch Scheduling Within a State 40‐Min Recess Policy
Source: J Sch Health. 2025 Aug 8;95(10):837–45. doi: 10.1111/josh.70055 (PMC12400478; doi:10.1111/josh.70055)
Supplement: Supplementary file 1 — Data S1. [file JOSH-95-837-s001.docx]

**Supplemental Material**

**Supplemental Table 1:** Lunch start times by grade level

| **Lunch Start Times** | **Kindergarten** | **1** | **2** | **3** | **4** | **5** | **6** |
| --- | --- | --- | --- | --- | --- | --- | --- |
| 10:00:00 AM | 2 | 0 | 1 | 0 | 0 | 0 | 0 |
| 10:15:00 AM | 0 | 1 | 0 | 0 | 0 | 0 | 1 |
| 10:30:00 AM | 16 | 6 | 2 | 1 | 2 | 0 | 0 |
| 10:45:00 AM | 24 | 10 | 5 | 5 | 3 | 1 | 0 |
| 11:00:00 AM | 9 | 23 | 11 | 11 | 4 | 2 | 1 |
| 11:15:00 AM | 7 | 7 | 12 | 4 | 6 | 3 | 2 |
| 11:30:00 AM | 3 | 10 | 11 | 14 | 14 | 8 | 2 |
| 11:45:00 AM | 0 | 0 | 12 | 13 | 4 | 2 | 0 |
| 12:00:00 PM | 3 | 6 | 5 | 10 | 13 | 9 | 0 |
| 12:15:00 PM | 1 | 1 | 2 | 3 | 10 | 7 | 3 |
| 12:30:00 PM | 0 | 2 | 2 | 2 | 3 | 7 | 2 |
| 12:45:00 PM | 0 | 0 | 0 | 0 | 3 | 1 | 1 |
| 1:00:00 PM | 0 | 0 | 0 | 0 | 0 | 0 | 1 |
